# Hepatitis delta: virological and clinical aspects

**DOI:** 10.1186/s12985-017-0845-y

**Published:** 2017-09-13

**Authors:** Luan Felipo Botelho-Souza, Mariana Pinheiro Alves Vasconcelos, Alcione de Oliveira dos Santos, Juan Miguel Villalobos Salcedo, Deusilene Souza Vieira

**Affiliations:** 1 Laboratório de Virologia Molecular - FIOCRUZ – RONDÔNIA, Rua da Beira, 7671 - BR 364, Km 3,5 Bairro Lagoa, CEP: 76812, Porto Velho, RO CEP: 76812–329 Brazil; 2 Ambulatório de Hepatites Virais, Fundação Oswaldo Cruz Rondônia e Centro de Pesquisa em Medicina Tropical – CEPEM, Avenida Guaporé, 215, anexo Hospital CEMETRON, Agenor M de Carvalho, Porto Velho, RO CEP: 76812-329 Brazil; 3 Programa de Pós-Graduação em Biologia Experimental – PGBioExp, Rodovia Br-364, KM 9, CAMPUS UNIR, Porto Velho, RO CEP: 76801-974 Brazil; 4 Instituto de Infectologia Emílio Ribas, Av. Dr. Arnaldo, 165 - Pacaembu, São Paulo, SP 01246-900 Brazil

**Keywords:** HDV, Virology, Clinical aspects

## Abstract

There are an estimated 400 million chronic carriers of HBV worldwide; between 15 and 20 million have serological evidence of exposure to HDV. Traditionally, regions with high rates of endemicity are central and northern Africa, the Amazon Basin, eastern Europe and the Mediterranean, the Middle East and parts of Asia. There are two types of HDV/HBV infection which are differentiated by the previous status infection by HBV for the individual. Individuals with acute HBV infection contaminated by HDV is an HDV/HBV co-infection, while individuals with chronic HBV infection contaminated by HDV represent an HDV/HBV super-infection. The appropriate treatment for chronic hepatitis delta is still widely discussed since it does not have an effective drug. Alpha interferon is currently the only licensed therapy for the treatment of chronic hepatitis D. The most widely used drug is pegylated interferon but only approximately 25% of patients maintain a sustained viral response after 1 year of treatment. The best marker of therapeutic success would be the clearance of HBsAg, but this data is rare in clinical practice. Therefore, the best way to predict a sustained virologic response is the maintenance of undetectable HDV RNA levels.

## Background

The hepatitis delta or D virus (HDV) was discovered in 1977 by Rizzetto et al. during an analysis of liver biopsies from patients chronically infected with the hepatitis B virus (HBV) and more severe liver damage [1]. Analysis of the hepatocytes using the technique immunofluorescence showed the presence of a specific antigen, which was named with the Greek letter δ and subsequently renamed the Delta antigen. Concomitantly, the authors also found specific antibodies against the delta antigen (anti-HDAg) in the serum of these patients [1]. Initially, they believed it was a new serological marker for HBV; however, studies with chimpanzees made the association between HBV and HDAg even clearer [2].

Later it was demonstrated that HBV infection associated with HDAg (HBV/HDV) did not develop in chimpanzees that previously presented titers of antibodies against HBsAg (anti-HBs). However, the rapid rise and persistence of HDAg was observed in chimpanzees who were chronically infected with HBV. It was suggested that HDAg could be a new marker of a transmissible pathogen, an HBV variant or even another viral agent that would need HBV’s helper functions [3]. In their seroprevalence study, Rizzetto et al. noted the presence of anti-HDAg in the serum of patients from Italy and from around the world, suggesting that HDAg was widely distributed [4–6].

The next discovery was that, besides the association of this new antigen with HBsAg, HDAg presented itself as complexed to a small RNA [7], showing the possible genetic material of these viruses, demonstrating that their genetic material was not dependent on the HBV genome. Therefore, HDV was known for containing the smallest genome among the known RNA viruses (approximately 1682 base pairs), resembling viruses that infect plants [8]. In 1986 HDV was described as the first animal virus identified with a circular RNA genome due to the similarity of nucleotides; circular RNAs had hitherto only been found in plant viruses. It was noted that the size and its secondary structures were similar to higher plant viroid and virusoid RNA, which suggested that HDV may have originated from the plant instead of the animal world [9]. Another unique feature of RNA HDV is the ability to self-cleave due to the presence of a ribozyme in the genomic and antigenomic sequence of its RNA, discovered in 1989, which is a sequence of about 85 nucleotides capable of self-cleavage and self-binding [10]. Ribozymes are also present in viroids, although ribozymes described from plant viruses were different from those found in HDV [11].

Despite the presence of the ribozyme, research continued to emerge about how HDV could replicate with a very small genome encoding only HDAg. It was assumed that HDV used HBV’s machinery and cellular functions to enable its replication [12]. Only in 1993 was an important study published by Taylor and Fu [13] for understanding the role of HBV and hepatocytes in the replication of HDV. Transcription of genomic into antigenomic viral RNA was observed along with the fact that this process was carried out by the host cells’ RNA polymerase II. It was believed that the rod shape of the viral RNA would be recognized as double stranded DNA by RNA Polymerase II [13–15]. The final replication step is the rearrangement of the genomic and antigenomic RNA of HDV which is catalyzed by its ribozymes, which cleave the product of the genome/antigenome transcription to form the circular structure of HDV RNA [13, 16].

## Classification

HDV was recognized by the International Committee on Taxonomy of Viruses (ICTV), as a new virus species from vertebrates, the only representative of the *Deltaviridae* family, *Deltavirus* genus [17]. Although HDV is quite similar structurally and in its mode of replication to phytopathogens, viroids and virusoids, it is sufficiently different to be assigned to a separate genus. HDV is commonly classified as a satellite virus of HBV, as it is based on the biological principle that HDV is incapable of infection in the absence of HBV [14, 18].

## Viral structure

**Fig. 1:**
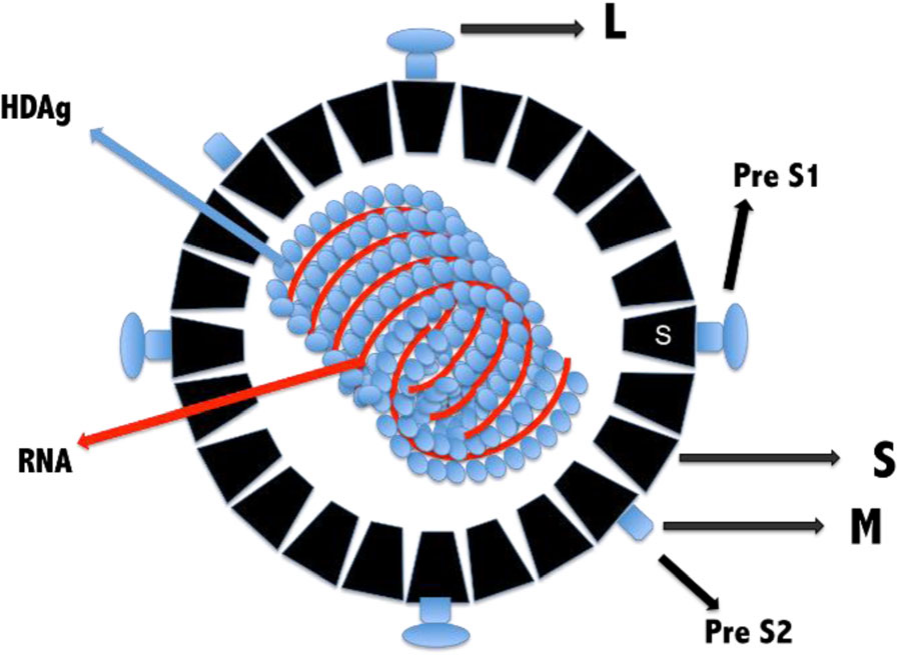
Schematic representation of HDV. The Hepatitis D virus wrapped in the HBV surface antigen. Outer HBsAg in black. With L (preS1 or large), M (preS2 or middle) and S (small) peripheral distribution of the three surface proteins. In the central portion, HDAg (HDAg-S and HDAg-L) is represented in blue and single-stranded RNA in red

HDV is formed, on the outside, by a spherical lipoprotein envelope containing HBsAg [19]. On the inside of the virion there is a ribonucleoprotein composed of the viral genome complexed to HDAg (Fig. 1) [8, 18, 20].

The viral genome consists of one single-stranded circular RNA (ssRNA) of about 1.7 kb and negative polarity. Due to the large amount of GC, nearly 74% of the nucleotides complement themselves intramolecularly, may fold into a secondary structure similar to a rod and are found complexed to HDAg [14, 18, 21, 22].

### Delta antigen

The Delta (HDAg) antigen is the only protein encoded by HDV, a phosphoprotein and can be found in two protein forms: a short one named HDAg-S and a large one called HDAg-L, with molecular weights of 24 kilodaltons (195 amino acids) and 27 kilodaltons (214 amino acids), respectively [23]. Studies have shown that HDAg-S promotes RNA replication while HDAg-L promotes HDV RNA enveloping to assemble the virion [21, 24–27].

**Fig. 2:**
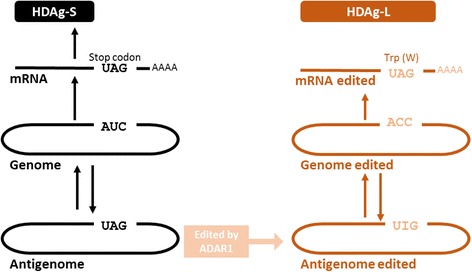
HDV RNA editing mechanism. Genomic RNA (gray rounded rectangle) serves as a template for mRNA synthesis which is translated into HDAg-S, which is necessary for synthesis of new RNAs. The genome serves as a template for the synthesis of the antigenome (rounded black rectangle), which also serves as a template for new genomic RNAs. A fraction of antigenomic RNAs are edited by the enzyme adenosine deaminase (ADAR-1) at the UAG site (stop codon), also called the Amber/W site (wavy and dotted arrow), wherein an adenine is replaced by inosine. The edited antigenomes serve as templates for the edited genomes (rounded rectangle edited for ACC). Edited genomes serve to synthesize edited messenger RNAs (with UGG – Tryptophan instead of UAG-stop codon) encoding HDAg-L, which is the key factor for enveloping the virus and inhibiting replication. The edited genome and antigenomes are simultaneously synthesized by a replication mechanism called rolling-circle. This way the editing levels accumulate replication products. Note that the numbering scheme is intended to indicate an increasing repertoire of activities that persist as replication occurs, rather than a gradual progression in which the above processes are terminated. (Source: [28]. Curr Top MicrobiolImmunol, our translation, modified)

During the replication cycle, the antigenome undergoes a post-transcriptional modification where the gene encoding HDAg-S is modified by an enzyme called Adenosine Deaminase (ADAR1), a host protein, substituting an adenine for an inosine, indirectly exchanging the UAG-stop codon for a UGG-tryptophan, known as the Amber/W site, which will give rise to the gene encoding HDAg-L with an additional 19 amino acids (Fig. 2) [28–31].

**Fig. 3:**
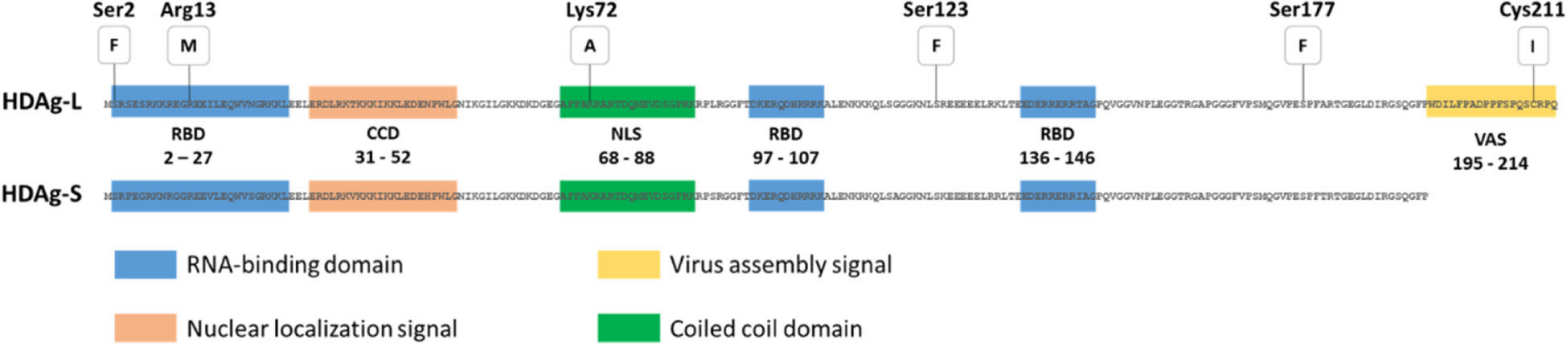
Structures of the two types of Delta antigen. (Source: [14], Journal of Virology, modified)

The difference between the two forms of HDAg is in the 19 additional amino acids in the C-terminal region of HDAg-L. Both HDAg isoforms have multiple functional domains in common, including the RNA-binding domain (RBD), a nuclear localization signal (NLS) a coiled coil domain (CCD) and a C-terminal portion of the sequence rich in proline and glycine. The 19 additional amino acids of HDAg-L are a virus assembly signal (VAS), which is a highly variable and specific sequence for each genotype [32]. Central to virus assembly, it serves as a binding site interacting with HBsAg/membrane (Fig. 3) [14].

HDAg undergoes several other post-transcriptional modifications such as phosphorylation [33, 34], acetylation [35] and methylation [36] and in the case of HDAg-L, isoprenylation [37]. The methylation of Arg13 [36, 38] acetylation of Lys72 [39], and phosphorylation of Ser177 [40] and Ser123 [41] were related to the subcellular localization of HDAg and RNA replication. Most of these changes are important for the functions of HDAg-S in HDV RNA replication which acts by directly stimulating the elongation of transcription through the substitution of the transcription repressor elongation factor linked to RNA polymerase II [42, 43].

## HDV RNA

**Fig. 4:**
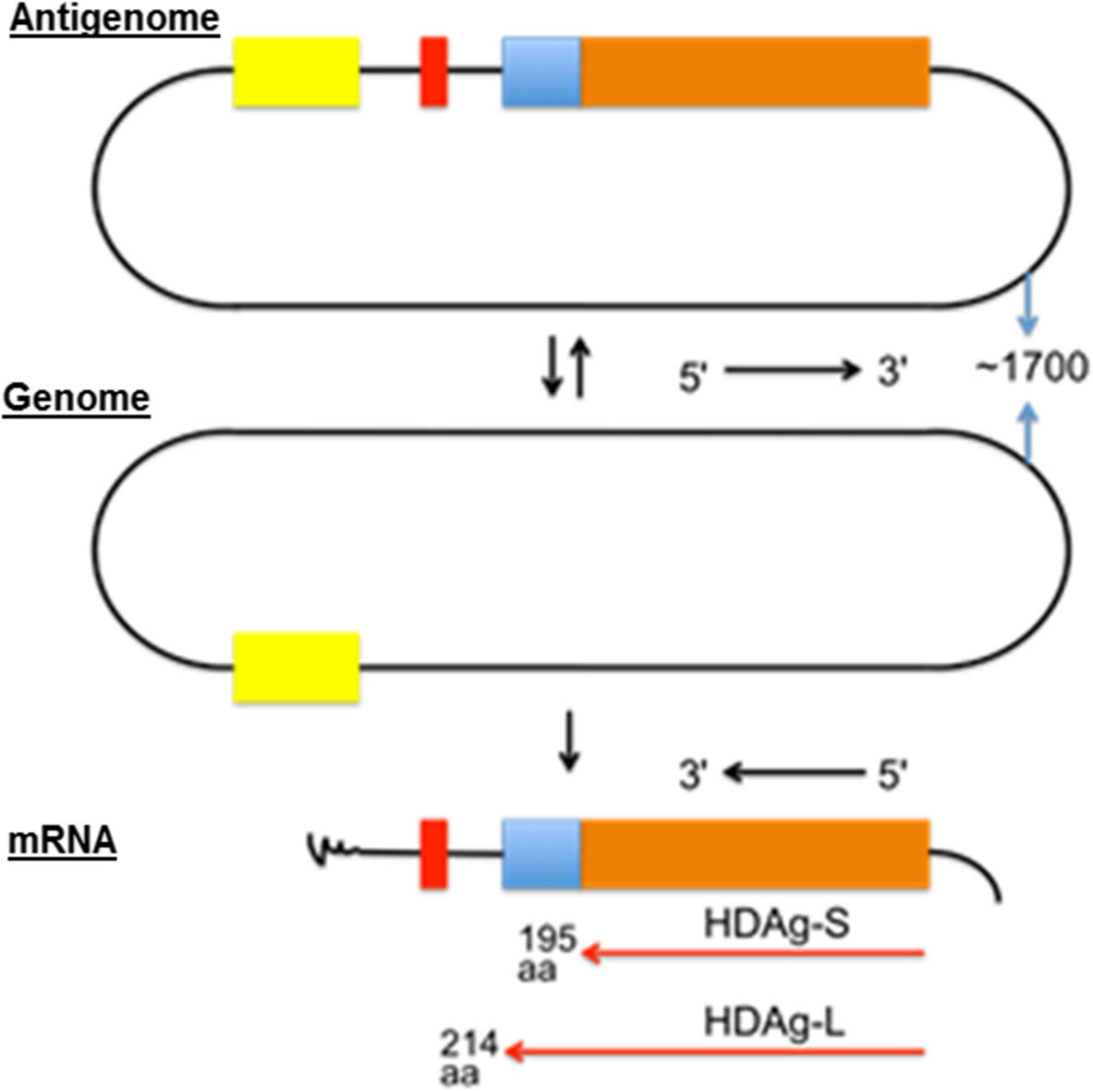
Schematic representation of HDV RNAs. Antigenome and genome of approximately 1700 nucleotides in a circular conformation (black). HDAg-S with 195 amino acids (orange) and HDAg-L with 214 amino acids (orange + blue). Negative polarity, the genome has an inverse orientation from the messenger RNA

Replication of the genome is completely directed by RNA, i.e., all the synthesis of new RNA has as its template HDV RNA itself, with no intermediate DNA template in the replication. In hepatocytes, HDV synthesizes complementary RNA, called antigenome, from its genome (Fig. 4) [32].

The genome and antigenome contain one ribozyme domain each with about 85 nucleotides [44, 45] which have the capacity to self-cleave and self-bind; this activity is an absolute requirement for viral RNA replication [46, 47].

## Viral replication

HDV uses a replication pathway called double-rolling circle, which is very similar to the strategy used by viroids, virusoids, and viroid-like satellite RNAs [48]. In general, the main feature of this type of replication is the use of a circular RNA strand as a template, which is transcribed by an RNA-dependent RNA polymerase of the host or helper virus. It is noteworthy that HDV is the only human pathogen that uses the host enzyme [49–51].

**Fig. 5:**
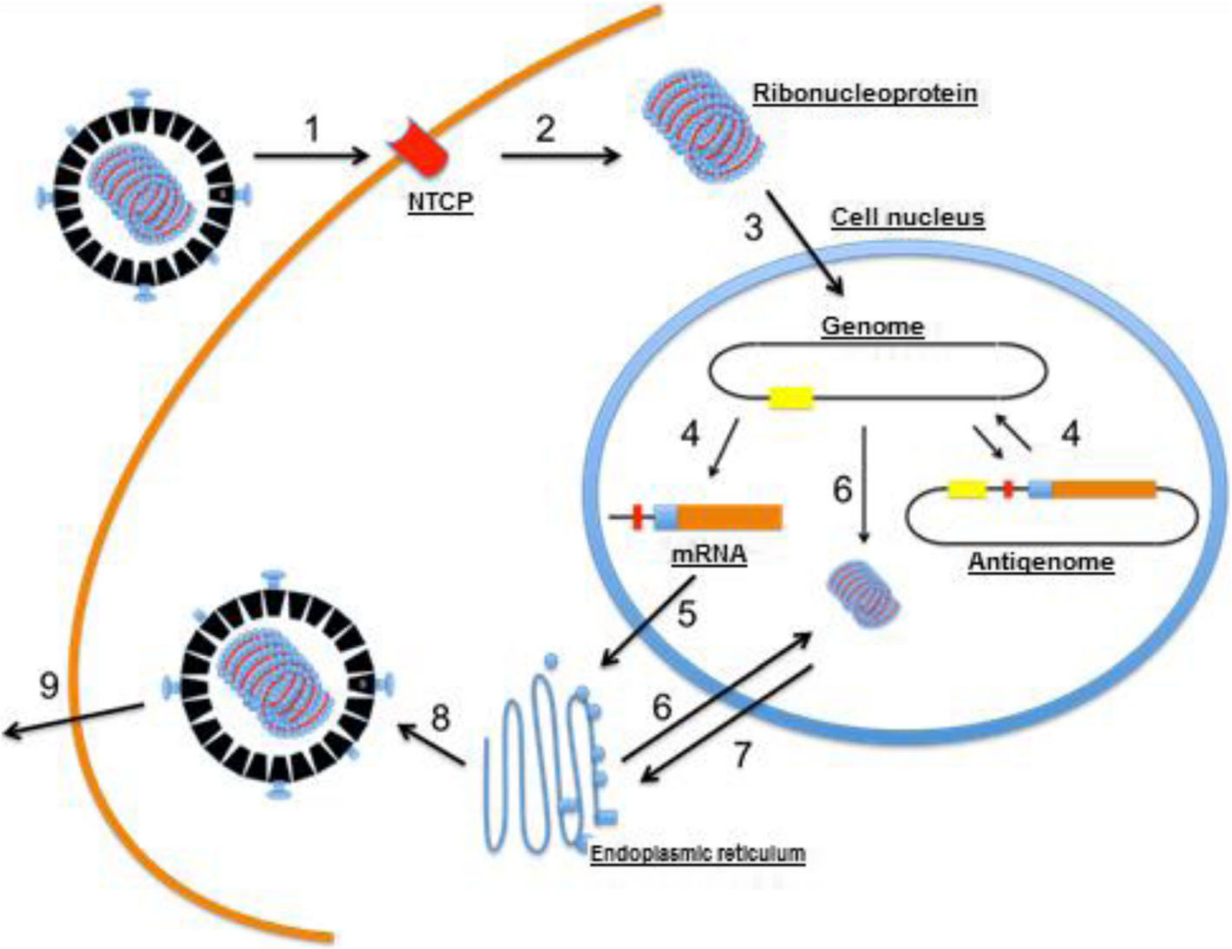
Replication and assembly cycle diagram. (1) The virion adheres to the hepatocytes via an interaction between HbsAg-L and a membrane receptor that has not yet been characterized, in the host cell, (2) the virion enters the cell and loses its envelope. (3) The ribonucleoprotein (HDV RNA complexed to HDAg) is imported into the nucleus of the cell, (4) the genomic RNA is transcribed in the nucleus into mRNA and antigenomic RNA, which in turn serves as a template for new RNA genomic transcripts. (5) The mRNA is exported to the cytoplasm where it is translated into HDAg-S in the endoplasmic reticulum, (6) the new HDAg-S molecules return to the cell nucleus to support the replication of more RNA. The two forms of HDAg associate themselves with the new genomic RNA to form new ribonucleoproteins, (7) which are exported to the cytoplasm where they will interact with HBV envelope proteins through HDAg-L in the endoplasmic reticulum to form new viral particles. (8) These particles by budding in an intermediate compartment (9) are exported from the hepatocyte via the trans-Golgi network to re-infect new cells. (Source: [20], Lancet, modified)

However, in the case of HDV where the host cell is a hepatocyte and due to the inexistence of RNA-dependent RNA polymerase in eukaryotic cells, in this replication the virus is deceptive and uses the hepatocyte’s own RNA polymerase. Subsequently, the new RNA strands suffer ribozyme-catalyzed cleavage and, finally, are connected by host cell enzymes (Fig. 5) [52].

In recent decades several studies have contributed to understanding HBV replication [53, 54] and suggest that the same mechanisms are involved in the initial process of hepatocyte infection. Initially, the surface proteins derived from the HBV envelope, would bind to heparan sulfate proteoglycans (HSPG). This connection, despite having low affinity [55], is important to the infectious process, since it helps in adhesion of the virion to its cellular receptor, the Na^+^ taurocholate co-transporting polypeptide (NTCP) [56]. Only after this virion [57–59] binding step with NTCP, high affinity binding occurs which will start the viral particle entrance process by endocytosis-mediated internalization [54, 60].

Upon entry into the cell, the HDV ribonucleoprotein is released into the cytoplasm and translocated by HDAg to the cell nucleus via the nuclear localization site (NLS); the ribonucleoprotein is imported by cellular importins [57–59].

In the nucleus, specifically in the nucleoplasm, the genomic RNA is transcribed by RNA polymerase II in unmodified mRNA that, in turn, migrates to the cytoplasm where it is translated into HDAg-S (required for replication of the HDV RNA) [13, 15, 55, 61]. In the nucleus, more specifically in the nucleosome, the genomic RNA is transcribed by RNA polymerase I [62] in a complementary RNA template, called antigenomic RNA. In the nucleoplasm, antigenomic RNA is transcribed by RNA polymerase II into new genomic RNA [61].

Later a fraction of antigenomic RNA undergoes editing by ADAR1 to serve as a template for edited genomic RNA; hence, the mRNA is edited and subsequently will give rise to HDAg-L [28]. Studies have shown that errors in the addition to polymerase and ADAR1-catalyzed amber/W editing, RNA recombination is also known can contribute to the genetic heterogeneity of HDV [51, 63, 64]. Some study show that recombination is not rare [65]. In the study realized by Sy et al. (2015), was show that can occur in the HDV genome for recombination located to the position nt908 in the genome which is downstream of the ribozyme activity (Rz) and polyadenylation signal sequence [66]. This recombination can contribuit for variability the genotypes HDV.

The two HDAg isoforms are sent on to the nucleus where they will be associated with the new unedited genomic RNA, forming a new ribonucleoprotein, which is exported to the cytoplasm [58]. Thus, it was observed clearly demonstrated that HDV RNA can replicate in hepatocytes and form HDV RNPs without the assistance of HBV [67]. However, HBV is indispensable for a productive HDV infection, considering that, in the assembly of the virion, HDAg-L will interact with HBsAg in the endoplasmic reticulum for viral involvement, thus producing an infectious viral particle, where, after there is the release of new viral particles through the Golgi complex, will be able to infect other cells [58, 68].

## Genotypes and epidemiology

The viral genetic diversity is related to the geographic origin of the isolates, and so far eight genotypes have been identified classified as HDV-1 through HDV-8 [69–72].

There are an estimated 400 million chronic carriers of HBV worldwide, between 15 to 20 million have serological evidence of exposure to HDV [72, 73]. Traditionally, regions with high rates of endemicity are central and northern Africa, the Amazon Basin, eastern Europe and the Mediterranean, the Middle East and parts of Asia [74]. HDV-1 is ubiquitous [75] and is often isolated in the United States, Europe and the Middle East, but has also been isolated in Russia, Africa, Asia and Brazil [24, 32, 71, 75–78] HDV-2, formerly known as genotype IIa, is found in Japan, Taiwan and Russia [79–81]. HDV-3 has been isolated in the Amazon region (Peru, Colombia, Ecuador and Brazil) [78, 82–85]. HDV-4 (the old genotype IIb) is found in Taiwan and Japan [80, 81, 86]. The genotypes HDV-5, HDV-6, HDV-7 and HDV-8 are found in Africa [69, 71]. HDV-8 was isolated in the countryside of the state of Maranhão (Brazil) in two native individuals [87].

HDV-3 is responsible for outbreaks of severe and common fulminant hepatitis in northeastern South America [76]. Studies show that HDV-3 is prevalent in the Brazilian Amazon. This genotype is apparently related to the more aggressive nature of HDV [76, 77, 84, 85, 88].

The western Amazon basin, including Brazil, Peru, Ecuador, Venezuela and Colombia, represents one of the highest rates of HBV infection in the world [89]. In Brazil, this area corresponds to the states of Acre, Amazonas, Rondônia and Roraima, with significant prevalence in the indigenous population [90, 91].

Braga et al. [92], in a study about the prevalence of HBV in Labrea, Amazonas, 11 years after the introduction of the HBV vaccine in the region, showed an HBsAg prevalence of 3.3% and an anti-HBc prevalence of 49.9%. However, in a study done previous to the introduction of the hepatitis B vaccine, the prevalence of HBsAg in this region of the Amazon was described as 16.7% [90]. Despite the prevalence of HBsAg having had a significant decline in the region, it is still considered a region with moderate endemicity for HBV.

In the state of Amazonas, the basins of the Juruá, Solimões and Purus rivers are areas of high endemicity for HBV and HDV, representing a major public health problem with serious cases, including fulminant hepatitis. A recent study in the region reported that almost a third (29.5%) of patients with HBV were co-infected with HDV [89]. Similar results were also described in Lábrea by Braga et al. [92]; of those who tested positive for HBsAg, the prevalence of anti-HDV was 30%. Later, Braga and colleagues described an anti-HDV prevalence of over 40% in chronic HBV carriers [93]. Fonseca obtained similar results, before the introduction of the hepatitis B vaccine, describing in the state of Amazonas an anti-HDV prevalence of 34.4% in patients chronically infected with HBV [90]. In Brazil, outside the Amazon region, few studies on the prevalence of HDV have been done, showing a very low prevalence.

In Brazil, studies show a significantly lower frequency in patients not co-infected with HIV than those co-infected with HIV, contrary to that described for other countries where prevalence can reach 50% in this population. A study about HDV prevalence in patients co-infected with HIV-HBV in the state of São Paulo, showed a rate of HDV of 1.2%, only one patient with anti-HDV was positive of the 81 with chronic hepatitis B. Similarly, in the state of Mato Grosso, in an analysis of 37 HBsAg positive patients only one was positive for HDV (2.7%). Corroborating data are described even in endemic areas, like in the Western Amazon, where a prevalence of 9.4% in patients with chronic hepatitis B was described. Although an increased prevalence was observed compared to the other two studies, it is significantly lower compared to the prevalence in the non-HIV population (34.4%) [94].

## Clinical aspects

**Fig. 6:**
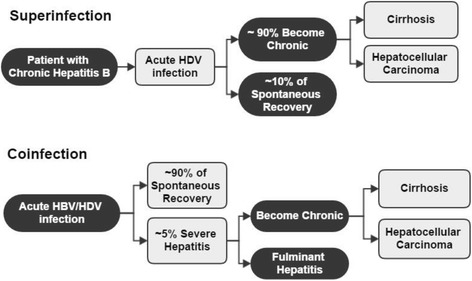
Schematic representation of the clinical course of hepatitis Delta

Due to the need for association with HBV, HDV can only be transmitted in the presence of a concomitant infection with HBV in one of two ways (super-infection or co-infection), depending on the previous status of the individual for HbsAg (Fig. 6) [95].

Co-infection is the simultaneous acute infection of HBV and HDV in a susceptible individual; this infection begins only after HBV has infected hepatocytes, and is similar to acute hepatitis B [96]. Because HBV is essential for HDV, the rate of progression to chronicity is similar to acute hepatitis B, i.e. between 2 and 5% [97, 98]. The incubation period of hepatitis D is dependent on HBV inoculum titers which determine the incubation time of hepatitis B [16]. Acute hepatitis may present itself as monophasic (one phase) and biphasic (two different phases), the first phase being dependent on HBV titers and the second on HDV titers [95]. In 95% of cases, spontaneous healing is seen, making it an important cause of severe or fulminant hepatitis [99].

Super-infection is an HDV infection in an individual chronically infected with HBV. It can cause fulminant hepatitis and chronicity rates are above 80%, which is related to an increased risk of early development of cirrhosis and hepatocellular carcinoma [1, 100, 101] It has a varied clinical appearance, although it generally causes a more severe acute hepatitis and has a relatively short period of incubation. In asymptomatic HBsAg patients it can lead to acute hepatitis, while in patients with chronic active hepatitis B, it can lead to exacerbation of symptoms, with liver decompensation. Patients who have a super-infection progress to chronic hepatitis in approximately 90% of cases [95].

HDV infection, whether in the form of a co-infection or a super-infection, is a significant cause of fulminant viral hepatitis, which is caused by HDV more often than by other forms of viral hepatitis [95]. A study conducted in Samara, Russia, showed that of the 27 diagnoses of fulminant viral hepatitis, 13 were infected with HDV, and of these 11 were males and 2 were females [102]. Some studies in different countries of South America show that acute hepatitis caused by an HBV and HDV co-infection or super-infection is the major cause of severe acute hepatitis in the Amazon region [84, 103].

According to a prospective analysis of 33 patients with chronic HDV admitted to a Spanish hospital in the period from 2006 to 2007, viral replication of HBV and HDV was quite variable during the study. In 54.5% HDV replication predominated, in 30.3% HBV predominated and in 15.2% HBV and HDV maintained similar levels. These data suggest that there is a suppression of HBV by HDV, but with important replication fluctuation of the two viruses [104]. The persistence of HBV replication, even at minimum levels, is associated with worse hepatocellular damage [105]. A third form has been described in patients after liver transplantation: latent infection [101]. Characterized by the presence of anti-HDV in the liver, hepatocyte nuclei in the absence of HBsAg and HDV RNA in the blood, is associated with low hepatocellular damage.

The chronic form of hepatitis D is the most severe and rapidly progressive of all chronic viral hepatitides. It leads to cirrhosis in approximately 70% within 5 to 10 years, usually involving patients of a young age. One to 2 years after the episode of acute hepatitis D, 15% develop cirrhosis. The risk of developing cirrhosis is 3 times higher in an HDV infection than in an HBV mono-infection [106]. A study in the Amazon region of Brazil showed that over 50% of treated patients treated with chronic hepatitis D who underwent a biopsy had moderate to intense fibrosis [91].

Fulminant hepatitis D has a dramatic evolution, with very poor prognosis. The clinical course occurs varying from 4 to 30 days after the onset of acute hepatitis D symptoms [107]. Transaminase levels may be high, but with massive liver necrosis, these levels tend to decrease rapidly. The same occurs with HDV replication levels, since there are few viable hepatocytes. If there is no liver transplantation in the first 10 days, mortality reaches approximately 80% [95].

## Diagnosis

**Fig. 7:**
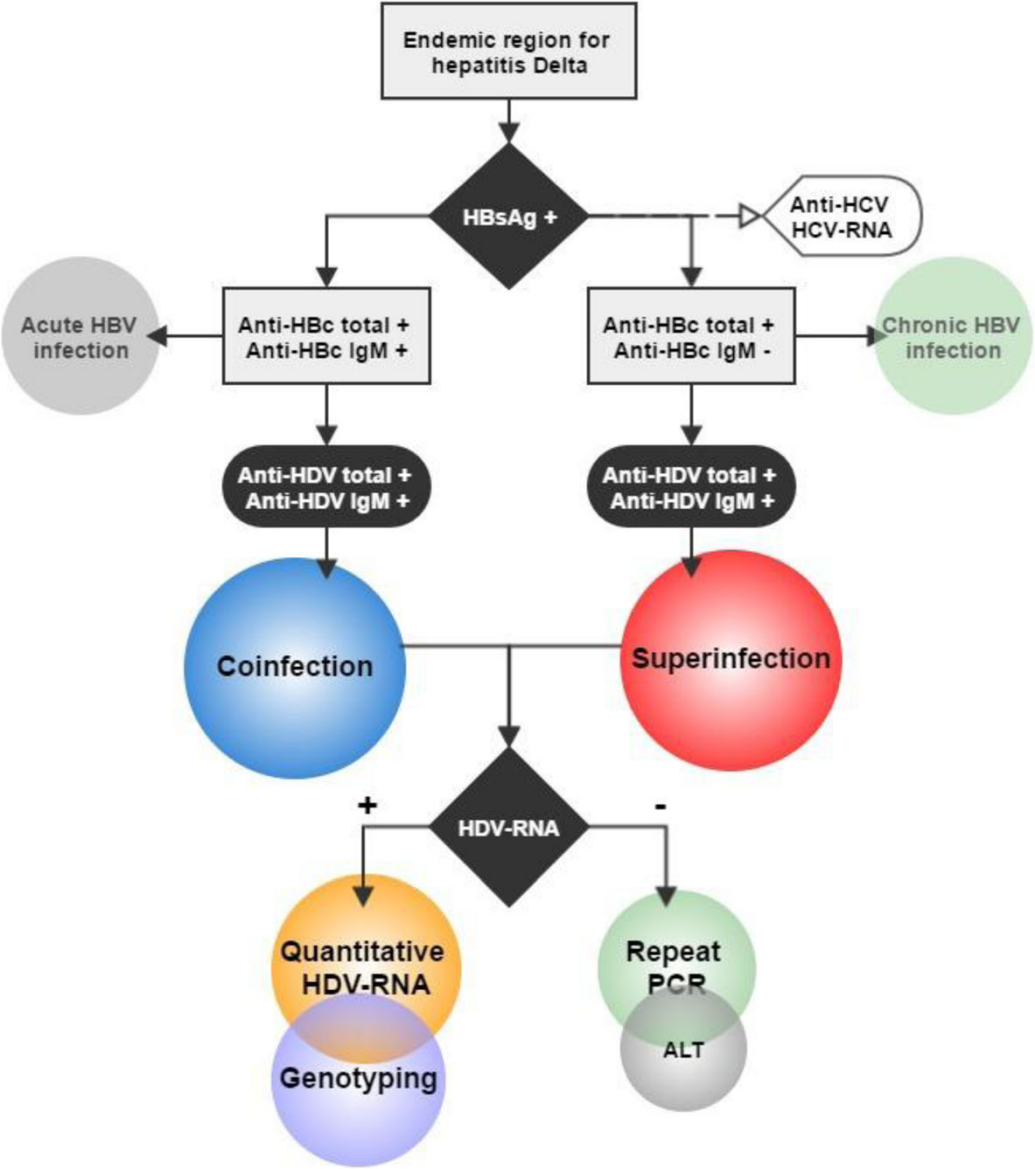
Fluxogram for the diagnosis of hepatitis Delta

The first step in the diagnosis of HDV is screening for antibodies against HDAg (anti-HDVIgM and IgG) in HBsAg positive individuals. In patients with the anti-HDV reagent, the next step is to screen for HDV RNA in the serum to determine whether the presence of the antibody against HDAg reflects a persistent active infection (HDV RNA positive) or only represents a decreasing serological scar (HDV RNA negative). In individuals with an HDV infection and liver disease (changes in Alanine aminotransferase (ALT), see Fig. 7) it is crucial to distinguish the type of HDV/HBV infection, whether it be an acute co-infection or super-infection in patients with chronic HBsAg, since the prognosis and management of the two types of infection are different [108, 109].

In individuals who have the reagent for HBsAg and live in areas endemic for hepatitis Delta (such as the western Amazon) it is recommended to determine the phase of HBV infection (acute or chronic) through screening for total anti-HBc and IgM. Acute HBV infection is characterized by the presence of anti-HBc IgM, and when accompanied by total anti-HDV and/or IgM reagents, it corresponds to an HDV co-infection. Chronic HBV infection is characterized by the presence of anti-HBc IgG (total anti-HBc reagent with non-reactive anti-HBc IgM), and when accompanied by total anti-HDV and/or IgM reagents it corresponds to an HDV super-infection (Fig. 7) [110, 111].

Both in an HDV co-infection and an HDV super-infection, screening for HDV RNA using molecular techniques such as polymerase chain reaction (PCR) is necessary, as the goal is to quantify the circulating virus in the blood (real time PCR) and determine the genotype (conventional PCR, restriction fragment length polymorphism - RFLP or sequencing) ^76,^ [112]^,119^. In the case that HDV RNA is negative it is recommended to repeat PCR and/or if necessary, the use of other diagnostic methods such as immunohistology for a liver biopsy and biochemical methods like verification of ALT. It is also important to screen for other viral infections such as the hepatitis C virus (HCV) by screening for anti-HCV and/or HCV RNA [113].

Liver biopsy is an important tool for the elucidation of the diagnosis of more than 20% of patients with unknown causes of cirrhosis [114]. Histologically acute hepatitis D is characterized by necrosis and hepatocellular inflammation, with lymphocytes and Kupffer cells infiltrating the parenchyma and portal region. This described pattern is not specific to hepatitis D, but despite being common to all forms of viral hepatitis, in hepatitis D it generally tends to be more intense [16]. Injury to the hepatocyte is typically focal, except in severe cases where confluent necrosis is seen [115]. In chronic hepatitis D, the histopathology consists of hepatocellular necrosis and inflammation in the parenchyma and portal region, associated with varying degrees of liver fibrosis [116]. Periportal necrosis is most commonly found in the other forms of viral hepatitis [95]. In patients with HDV, the definitive diagnosis by liver biopsy is done by identifying HDAg [114]. The amount of HDAg decreases with the progression of fibrosis, being almost undetectable in the final stage of the disease [16].

## Treatment

The appropriate treatment for chronic hepatitis delta is still widely debated because of the lack of an effective drug. Alpha interferon is currently the only therapy licensed for the treatment of chronic hepatitis D [117, 118]. The most used drug is pegylated interferon, but only about 25% of patients maintain a sustained viral response after 1 year of treatment [119]. Transaminase levels normalize in only 40–70% of treated patients and relapse occurs in 60–97% [120]. A sustained virologic response is described as occurring when the viral load is negative 6 months after the end of treatment [119].

Some studies were developed in an attempt to find a more effective therapy than Interferon monotherapy. However, these studies were not encouraging showing that monotherapy with lamivudine, entecavir, adefovir, tenofovir, famciclovir and ribavirin or combination therapy with interferon and a nucleos (t) ide analog was no more effective than monotherapy with Interferon [121–124].

The duration of therapy is also controversial but some studies have shown that 2 years of treatment did not obtain better results than those treated for 1 year [125, 126]. Patients with HDV viremia and elevated transaminases should initiate treatment with Peg-interferon alpha 2a or 2b for 1 year. In cases where HDV RNA is negative, treatment should be terminated and the patient monitored. However, in cases where after 1 year of therapy there still remain detectable levels of HDV RNA, another year of therapy is indicated, especially with elevated transaminase levels [119].

In patients with decompensated cirrhosis, the use of interferon is contraindicated, but in patients with compensated cirrhosis, peg-interferon was effective [127].

Later studies suggest that the virologic response was not altered by previous treatment with interferon, with similar results being described in two major studies [125, 128], with virologic response rates sustained between 20 and 25%, both in naive patients as well as in those being re-treated.

Alternative treatment options are currently being explored in clinical trials, prenylation inhibitors are promising and has been assessed in patients with chronic HDV infection. Lonafarnib showed a dose-dependent reduction of HDV RNA levels in patients after 28 days of therapy [129].

With recent description of the receptor-mediated entry HBV, also used by HDV, novel therapeutic strategies for treatment of Delta hepatitis was developed that interfer this entry. Blockade of the HBV-HDV-specific receptor sodium/taurocholate cotransporting polypeptide NTCP by Myrcludex show promising results [130]. The HBV entry inhibitor myrcludex B is also being developed. Bogomolov and cols described a myrcludex B in patients with chronic hepatitis D infection either alone or in combination with Peg-INF alpha 2a as compared to patients only treated with Peg-INF alpha 2a [131]. Myrcludex B monotherapy was associated with HDV RNA and HBV DNA declines and improvement of ALT levels after 24 weeks of treatment. The antiviral effect was more pronounced en combination with Peg-INF alpha 2a [131].

Preliminary results of 6 months treatment with a subcutaneous HBV PreS1-derived myristoyled peptide as an entry inhibitor indicates an encouraging short-term response with low side-effects [132].

Likewise, the observation that HDV assembly requires farnesyltransferase, have enabled another novel therapeutic strategies. Short-term use of oral farnesyl transferase inhibitors induces a log10 reduction of viral RNA in almost all treated patients, but is associated with gastrointestinal upset and weight loss (especially using 200 mg/day) [133].

Recent studies comparing the antiviral effects of Peg-INF alpha, Peg-INF lambda and entecavir in human liver chimeric mice demonstrated that Peg-INF alpha and Peg-INF lambda reduced HDV viremia, serum HBsAg levels and intrahepatic levels of genomic and antigenomic HDV RNA [134].

Many treatment protocols have been tested worldwide (Table 1). Knowledge, Borzacov and colleagues [135] reported in a clinical study as better virologic response at the end of treatment (95%) and sustained virologic response (95%) for the treatment of delta hepatitis.

**Table 1:** Tested treatment protocols

Drug/Dose	N	Duration	Follow-up	VRET	SVR	Study
IFN α2a 9MU/m 3×/wk	14	48 wks	6 months	71%	43%	Farci, 1994 [145]
IFN α2a 3MU/m 3×/wk	14	48 wks	6 months	36%	14%	
Placebo	14	48 wks	6 months	0%	8%	
IFN α2b 18MU 3×/wk. +	16	6 months	18 months	31%	–	Madejon, 1994 [146]
*9MU 3×/wk. +*		*1 month*				
*6MU 3×/wk. +*		*1 month*				
*3MU 3×/wk*		*4 months*				
IFN α2a 3MU/day +	16	3 months	18 months	25%	–	
1.5MU		9 months				
Ribavirin 15 mg/kg/day	9	4 months	12 months	11%	11%	Garripoli, 1994 [147]
Famciclovir	15	6 months	6 months	6.60%	13.30%	Yurdaydin, 2002 [148]
Lamivudine 100 mg/day + Placebo	11	52 + 52 wks	16 wks	9.0%	0%	Niro, 2005 [121]
Lamivudine 100 mg/day	20	104 wks	16 wks	10%	10%	
INF α 2a 9MU 3×/wk.	10	24 months	6 months	50%	20%	Günsar, 2005 [125]
INF α 2a 9MU 3×/wk. + Ribavirin 1–1,2 g/day	21	24 months	6 months	52%	23%	
Peg-INF α 2b 1.5 μg/kg/wk	14	12 months	16 months	57%	43%	Castelnau, 2006 [149]
Peg-INF α 2b 1.5 μg/kg/wk	12	12 months	12 months	–	17%	Erhardt, 2006 [122]
Peg-IFN α 2b 1.5 μg/kg/wk.	16	18 months	6 months	19%	25%	Niro, 2006 [150]
Peg-IFN α 2b 1.5 μg/kg/wk. + Ribavirin 800 mg/day +	22	12 months	6 months	9%	18%	
Peg-IFN α 2b 1.5 μg/kg/wk.		6 months				
INF α 2b 10MU 3×/wk	12	12 months	24 months	42%	17%	Canbakan, 2006 [151]
INF α 2b 10MU 3×/wk. + Lamivudine	14	12 months	24 months	64%	28%	
IFN α 2a 9MU 3×/wk	8	12 months	6 months	50%	50%	Yurdaydin, 2008 [152]
Lamivudine +	14	2 months	6 months	50%	36%	
IFN α 2a 9MU 3×/wk. + Lamivudine		10 months				
Lamivudine	17	12 months	6 months	12%	12%	
Peg-INF α 2b 1.5 μg/kg/wk	49	13 months	26 months	33%	25%	Gheorge, 2011 [128]
Peg-INF α 2b 1.5 μg/kg/wk	11	24 months	6 months	56%	–	Örmeci, 2011 [153]
	7	12 months	6 months	57%	–	
Peg-IFN α 2b 180 μg/wk. + Adefovir 10 mg/day	31	12 months	6 months	23%	26%	Wedemeyer, 2011 [154]
Peg-IFN α 2b 180 μg/wk.	29	12 months	6 months	24%	31%	
Adefovir 10 mg	30	12 months	6 months	0%	0%	
Entecavir 1 g/day	13	48 wks	6 months	23%	–	Kabaçam, 2012 [124]
Peg-IFN α 2a 180 μg/wk. ou 2b 1.5 μg/kg/wk	32	24 months	6 months	50%	47%	Karaca, 2013 [155]
Peg-IFN α 2b 180 μg/wk. + Entecavir	22^a^	12 months	6 months	95%	95%	Borzacov, 2016 [135]

Notes: *IFN* Interferon, *N* Number of patients, *VRET* Virological response at end of treatment, *SVR* Sustained virologic response

^a^all genotype 3

### Therapeutic response markers

The best marker of therapeutic success would be the clearance of HBsAg, but this data is rare in clinical practice [136]. Therefore, the best way to predict the sustained virologic response is the maintenance of undetectable HDV RNA levels [119]. However, therapeutic success may be related to the decrease in HDV RNA and HBsAg levels and the normalization of ALT; even without its undetectability, it is associated with reduced events related to liver disease when treated with IFN [135, 137]. The treatment showed, in most studies, a low percentage of undetectability and a high rate of relapse, even if it was late. An early virological response, i.e., undetectable HDV RNA or a 3 log decrease during the first 6 months of treatment, may be indicative of a sustained virologic response [122, 139, 142].

## Hepatocellular carcinoma

The increased frequency of cirrhosis associated with increased induction of liver inflammation in chronic carriers of HDV represent indirect risk factors for HCC [138]. However, a further increase in oncogenicity due to HDV infection itself has not been proven [139, 140].

Some studies suggest that an HBV/HDV co-infection increases the risk of HCC [140], showing a 3-fold increase in the risk of HCC and 2 times the mortality compared to patients with an HBV mono-infection [106, 141]. However, a retrospective analysis of 962 patients with HBV, 82 of whom were co-infected with HDV, showed similar rates of HCC in both groups [142]. Thus, this relationship is still controversial in the literature and the role of HDV in the induction and development of HCC should be further studied.

## Prognosis

An Italian study conducted in Milan analyzed 299 patients with chronic hepatitis D admitted between 1978 and 2006; the probability of survival 20 years after the diagnosis of chronic HDV infection was 86%. The persistent replication of HDV was the only predictive factor associated with an increased risk of mortality [98]. However, quantitative values of HDV RNA do not seem to be correlated with the degree of liver disease in patients infected with HDV [143].

Farci et al., in a longer follow-up, on average 13 years, of patients previously treated with IFN alpha 2a 9MU (*n* = 14), 3MU (n = 14) and the control group (n = 14) showed that, although no patients had negated the viral load, survival was significantly higher in those who used high IFN doses, with no significant difference between those who used low doses and the controls. A significant improvement in necroinflammatory lesions was also observed in the liver biopsy of patients treated with high doses. In this study, there is an estimated survival in 12 years after treatment, without the need for transplantation, of 86% among those who received 9MU, 39% with 3MU and 31% in the control group [135].

A recent cohort study shows that anti-HDV IgM may persist in patients with chronic hepatitis from HDV or reappear in who have relapsed after treatment. It may be associated with increased levels of ALT and bilirubin and decreased levels of albumin, as well as histological activity [144].

## Conclusion

This review of the clinical and virological aspects of hepatitis D demonstrates strategies for the diagnosis and treatment of the disease. Unfortunately, the lack of an effective anti-HDV drug directs therapy for pegylated interferon, but only a small percentage maintains the virological response after 1 year of treatment. Therefore, therapeutic success may be related to decreased levels of HDV and HBsAg and normalization of ALT, however, even without its undetectability, it is associated with reduced events related to liver disease when treated with pegylated interferon.

## Abbreviations

ALT: Alanine aminotransferase
anti-HDAg: Antibody produced in our body to fight the antigen (HDAg)
CCD: Coiled coil domain
HBsAg (anti-HBs): Antibody produced in our body to fight the antigen (HBsAg)
HBsAg: Hepatitis B virus surface antigen
HBV: Hepatitis B virus
HCC: Hepatocellular carcinoma
HCV: Hepatitis C virus
HDAg: Antigen Delta
HDAg-L: Protein large antigen Delta
HDAg-S: Protein short antigen Delta
HDV: Hepatitis D virus
HIV: Human immunodeficiency virus
HSPG: Heparan sulfate proteoglycans
ICTV: International Committee on Taxonomy of Viruses
NLS: Nuclear localization signal
NLS: Nuclear localization site
NTCP: Na^+^ taurocholate co-transporting polypeptide
PCR: Reaction chain polymerase
RBD: RNA-binding domain
RFLP: Restriction fragment length polymorphism
RNA: Ribonucleic acid
ssRNA: Single-stranded circular RNA
